# The effects of cyclooxygenase-2 gene silencing by siRNA on cell proliferation, cell apoptosis, cell cycle and tumorigenicity of Capan-2 human pancreatic cancer cells

**DOI:** 10.3892/or.2011.1595

**Published:** 2011-12-19

**Authors:** YINGQIANG ZHONG, ZHONGSHENG XIA, JUAN LIU, YING LIN, HUI ZAN

**Affiliations:** 1 Department of Gastroenterology, Sun Yat-Sen Memorial Hospital, Sun Yat-Sen University, Guangzhou 510120; 2 Department of Gastroenterology, The Affiliated Hospital of North Sichuan Medical College, Nanchong 637000, P.R. China

**Keywords:** pancreatic neoplasm, cyclooxygenase-2, RNA interference, cell proliferation, apoptosis, cell cycle

## Abstract

The prognosis of pancreatic cancer is still very poor. No specific effective gene therapy for pancreatic cancer has been found. As a key enzyme of the metabolic process of arachidonic acid, cyclooxygenase-2 (COX-2) has been found to be closely related to the tumorigenesis of epithelial cancers. However, the antitumor effect of small interfering RNA (siRNA) targeting COX-2 in pancreatic cancer has not yet been verified. Therefore, the aim of this study was to investigate the effects of COX-2 gene silencing by siRNA on cell proliferation, cell apoptosis, cell cycle and tumorigenicity of pancreatic cancer cells. COX-2 mRNA was detected by RT-PCR and real-time PCR. COX-2 protein was detected by Western blotting. The cell proliferation was measured by cell counting using microscopy. The cell apoptosis and cell cycle were measured by flow cytometry. The tumorigenicity of Capan-2 pancreatic cancer cells transfected with COX-2 siRNA was evaluated using a nude mouse xenograft model. The expression of COX-2 mRNA as well as COX-2 protein were downregulated after COX-2 siRNA transfection. COX-2 siRNA could inhibit the growth of Capan-2 cells significantly by decreasing the cell proliferation, increasing cell apoptosis and regulating cell cycle as well. *In vivo* experiments demonstrated that the mean volume and weight of subcutaneous xenografts in nude mice derived from Capan-2 cells transfected with COX-2 siRNA were significantly decreased. COX-2 siRNA could inhibit the growth of Capan-2 pancreatic cancer cells and also decrease the tumorigenicity of Capan-2 cells, implicating a new potential therapeutic target in pancreatic cancer.

## Introduction

The morbidity of pancreatic cancer has been increased globally in recent years. Moreover, the prognosis of pancreatic cancer is very poor. With the development of research in molecular biology, oncology and the related subjects, the gene therapy of pancreatic cancer is regarded as a new mode of cancer treatment subsequently following traditional therapy such as surgical operation, radiotherapy and chemotherapy. Cyclooxygenase-2 (COX-2), is a key enzyme of the metabolic process of arachidonic acid, which plays an important role in tumorigenesis, apoptosis and angiogenesis (1). Our previous study found that COX-2 was highly expressed in pancreatic cancer tissue and associated with other genes regulating cell growth of pancreatic cancer cells (2–4). Therefore, COX-2 is implicated as one of the molecular targets of gene therapy for pancreatic cancer. RNA interference (RNAi) is a post-transcriptional gene silencing process. RNAi is initiated by the enzyme Dicer which cleaves long double-stranded RNA (dsRNA) molecules into short fragments of 21–25 nt. These short double-stranded fragments are called small interfering RNAs (siRNAs). These siRNAs are then separated into single strands and integrated into an active RNA-induced silencing complex (RISC). After integration into the RISC, siRNAs base-pair to their target mRNA and induce cleavage of the mRNA, thereby leading to post-transcriptional gene silencing. Artificial synthetic siRNAs have become an important tool for research on gene function after gene silencing. siRNAs could effectively and specifically suppress the target gene expression, which implicate their application in the treatment of many refractory diseases including pancreatic cancer. In this study, we investigated the effects of COX-2 gene silencing by siRNA on cell proliferation, cell apoptosis, cell cycle and tumorigenicity of Capan-2 human pancreatic cancer cells. This study may provide experimental evidence for gene therapy in pancreatic cancer.

## Materials and methods

### Materials and reagents

COX-2 siRNAs labeled by fluorescence Cy3 (Cy3-siRNAs) were synthesized by Guangzhou Ruibo Biotechnology Co., Ltd. (Guangzhou, China). The RPMI-1640 medium, 10% FBS and Lipofectamine 2000 (Lipo) were obtained from the Gibco-Invitrogen Corporation (USA). The cDNA synthesis kit was obtained from Dalian Bao Bioengineering Co., Ltd. (Dalian, China). COX-2 mAb was purchased from the Cayman Chemical Corportation (USA). β-actin mAb was purchased from the Shanghai Kangcheng Biotechnology Corporation (Shanghai, China). HRP-conjugated goat anti-mouse IgG was purchased from the Shanghai Lianke Biotechnology Corporation. ECL™ Western Blotting Detection system was obtained from Thermo Corporation (USA). BCA™ protein assay kit was purchased from Shanghai Shenneng Lottery Biotechnology Corporation (Shanghai, China). Annexin V FITC/PI assay kit was purchased from Invitrogen Corporation (USA).

### COX-2 siRNA sequences

The COX-2 siRNA001, target sequence was 5′-GCTGGGAAGCCTTCTCTAA-3′ the sense strand was 5′-GCUGGGAAGCCUUCUCUAAdTdT-3′ and the antisense strand was 5′-dTdTCGACCCUUCGGAAGAGAUU-3′. COX-2 siRNA002, target sequence was 5′-GCAGCTTCCTGATTCAAAT-3′ the sense strand was 5′-GCAGCUUCCUGAUUCAAAUdTdT-3′ and the antisense strand, 5′-dTdTCGUCGAAGGACUAAGUUUA-3′. The COX-2 siRNA003, target sequence was 5′-GAATCATTCACCAGGCAAA-3′ the sense strand was 5′-GAAUCAUUCACCAGGCAAAdTdT-3′ and the antisense strand was 5′-dTdTCUUAGUAAGUGGUCCGUUU-3′. The COX-2 siRNA004, target sequence was 5′-AACACCGGAATTTTTGACAAG-3′ the sense strand was 5′-AACACCGGAAUUUUUGACAAGdTdT-3′ and the antisense strand, 5′-dTdTUUGUGGCCUUAAAAACUGUUC-3′. The COX-2 siRNA005 target sequence was 5′-GATTGAAGATTATGTGCAA-3′ the sense strand was 5′-GAUUGAAGAUUAUGUGCAAdTd-3′ and the antisense strand was 5′-dTdTCUAACUUCUAAUACACGUU-3′. The COX-2 siRNA006, target sequence was 5′-GGACTTATGGGTAATGTTA-3′ the sense strand was 5′-GGACUUAUGGGUAAUGUUAdTdT-3′ and the antisense strand was 5′-dTdTCCUGAAUACCCAUUACAAU-3′.

### PCR primers

PCR primers were synthesized by the Shanghai Yingjun Corporation (Shanghai, China). The RT-PCR primers were, COX-2 primers, forward, TTCAAATGAGATTGTGGGAAAAT and reverse, AGATCATCTCTGCCTGAGTATCTT. β-actin primer, forward, AAGGAAGGCTGGAAGAGTGC and reverse, CTACAATGAGCTGCGTGTGG. The real-time PCR primers were COX-2 primers, forward, CTGGAACATGGAATTACCCAGTTTG and reverse, TGGAACATTCCTACCACCAGCA; β-actin primers, forward, TGGCACCCAGCACAATGAA and reverse, CTAAGTCATAGTCCGCCTAGAAGCA.

### Cell culture

The Capan-2 human pancreatic cancer cell line was provided by the California University and the cells were cultured in RPMI-1640 medium supplemented with 10% FBS without addition of antibiotics at 37°C in a humidified incubator containing 5% CO_2_.

### siRNA transfection

Capan-2 cells were seeded at 1×10^6^/well in 6-well plates 1 day prior to transfection and 1.5 ml of medium without antibiotics was added into each well so that the cells grew to 30–50% confluence when the transfection was performed. The siRNA-Lipo mixture was prepared according to the manufacturer's instructions. The siRNA-Lipo mixture was uniformly added into each well containing cells and medium. The plate was shaken back and forth for mixing. The cells were then cultured at 37°C in a humidified incubator containing 5% CO_2_, and were collected after 24, 48 and 72 h of culture, respectively. Total-RNA was isolated by using the TRIzol reagent. Total protein was extracted by the RIPA assay. The RNA samples and protein samples were stored at −30°C.

### Screening test of Cy3-siRNAs and Lipo for transfection efficiency

To test the transfection efficiency of Lipo at different concentrations, 50 nM of Cy3-siRNAs and different concentrations of Lipo including 3, 4, 5 and 6 μl of Lipo were used. To test the transfection efficiency of Cy3-siRNAs at different concentrations, 5 μl of Lipo and different concentrations of Cy3-siRNAs including 30, 50 and 100 nM of Cy3-siRNAs were used.

### Screening efficient sequences of COX-2 siRNAs by using RT-PCR and real-time PCR

Single stranded cDNA was synthesized from 1 μg RNA using a cDNA synthesis kit. The conditions for reverse transcription were as follows: 65°C for 5 min, then 42°C 30 min and 95°C for 5 min to inactivate the enzyme. The newly synthesized cDNA was amplified by PCR using the GeneAmp2700 PCR instrument (GeneAmp, Germany). Cycling conditions were as follows: 94°C for 2 min to denature cDNA and primers, then followed by 30 cycles at 94°C for 30 sec, 55°C (COX-2) or 61°C (β-actin) for 30 sec and 72°C for 1 min. Real-time PCR was performed by using LightCycler 480 real-time PCR instrument (Roche, Switzerland). Cycling conditions for real-time PCR were as follows: 95°C for 30 sec to denature cDNA and primers, followed by 40 cycles at 95°C for 5 sec and 60°C for 20 sec.

### Western blotting

Protein expression of COX-2 was measured by Western blotting. β-actin was used as an internal control. Cellular proteins were dissolved in sample loading buffer and run on 7.5% sodium dodecyl sulfate-polyacrylamide gel electrophoresis (SDS-PAGE) gels (100 V, constant voltage, 60 min). COX-2 protein was electrotransferred onto PVDF membranes (4°C, 200 mA, 100 min). The membranes were rinsed with PBS and blocked with 10% nonfat milk in PBS for 3 h at room temperature. Membranes were then incubated with the primary antibody of COX-2 (1:1,000) or β-actin (1:5,000) in 3% nonfat milk overnight at 4°C. After primary antibody incubation, membranes were rinsed in TBS-T wash buffer for 10 min 3 times each. Membranes were then incubated with secondary antibody (HRP-conjugated goat anti-mouse IgG, 1:5,000) for 2 h at room temperature and rinsed in TBS-T wash buffer for 10 min 3 times each. The protein-antibody complexes were visualized by ECL™ Western Blotting Detection system.

### Experiment grouping in vitro

Capan-2 human pancreatic cancer cells were cultured in 6-well plates. The efficient COX-2 siRNA was transfected into Capan-2 cells. Capan-2 cells were harvested respectively at 24, 48 and 72 h after transfection. Cell counting and flow cytometry (FCM) were performed. Capan-2 cells without transfection were set as blank control group. Capan-2 cells treated with negative siRNA transfection were set as negative control group. Capan-2 cells treated with only Lipo were set as the liposome control group.

### Cell proliferation analysis

Cell proliferation analysis was performed by the cell counting method. Capan-2 human pancreatic cancer cells were seeded at 2×10^5^ cells/well in 6-well plates. After 24 h incubation, siRNA transfection was performed. Subsequently, cells were incubated at 37°C in 5% CO_2_. Cells were harvested by digestion of 0.25% trypsin at 24, 48 and 72 h respectively after transfection. Cells of each well were counted three times. Cell growth curves were drawn based on the average cell count of each group.

### Analysis of cell apoptosis and cell cycle

Capan-2 human pancreatic cancer cells were seeded at 2×10^5^ cells/well in 6-well plates. After 24 h incubation, siRNA transfection was performed. Then cells were incubated at 37°C in 5% CO_2_. Cells were harvested at 24, 48 and 72 h respectively after transfection and washed twice with cold PBS. Cells were resuspended with 1 ml of PBS to be 1–5×10^6^/ml of cell suspension and 0.5 ml of cell suspension was used for cell apoptosis analysis. The remaining portions were used for cell cycle analysis. Analysis of cell apoptosis and cell cycle were performed by FCM using the FACSCalibur™ flow cytometer (Becton-Dickinson, San Jose, CA).

### Experiment in vivo

Animal care and euthanasia were approved by the Sun Yat-Sen University animal studies committee. Eighteen BALB/c nu/nu nude mice (experimental animal center, Sun Yat-Sen University, China) were randomly divided into three groups including the control group, negative control group and COX-2 siRNA group. Each group consisted of 6 nude mice. As the most efficient COX-2 siRNA, COX-2 siRNA006 was selected for transfection *in vivo*. Cells including parent Capan-2 cells, Capan-2 cells transfected with negative siRNA and Capan-2 cells transfected with COX-2 siRNA006 were harvested in the exponential growth phase and washed with cold PBS for three times. The cells were resuspended with PBS to a 0.2 ml single cell suspension (5×10^6^ cells/ml). The cell suspensions were inoculated subcutaneously into flanks of 4–6-week-old BALB/C nu/nu nude mice. Xenografts in each group were observed periodically after inoculation. The tumorigenicity of xenografts was evaluated. The length and width of xenografts were measured. The volume of the xenograft was calculated according to the following formula: V=ab^2^/2 (V, volume; a, length; b, width). The inhibition rate of the xenograft was calculated based on the average volume of the xenograft. Nude mice were sacrificed by cervical dislocation at 6 weeks after inoculation. The xenografts were peeled off subcutaneously. The weight of the xenografts in each group was compared.

### Statistical analysis

All measurement data were present as mean ± standard deviation (SD). The differences among multiple mean values were evaluated using analysis of variance (ANOVA). The differences between two mean values were estimated using independent-samples t-test. Values for counting data were present as a rate or ratio. The differences among the groups were analyzed using the χ^2^ test. All of the statistical analyses were processed with the statistical analysis software SPSS, version 10.0 (SPSS, Chicago, IL).

## Results

### Transfection efficiency and the most optimal transfection concentration of Lipo and siRNA

**Figure 1:**
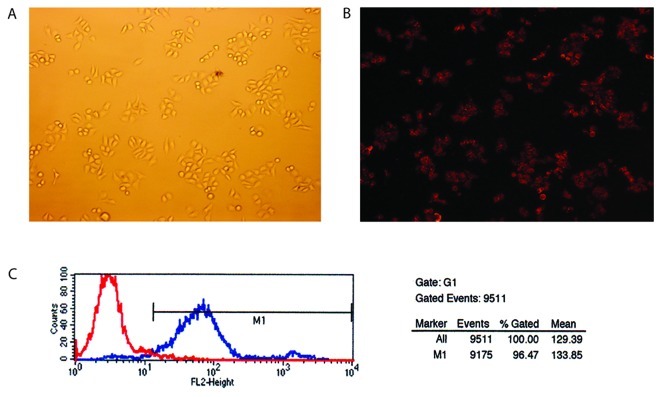
Transfection efficacy was assessed by Cy3-siRNA (x100) and FCM (flow cytometry). (A) Common light microscopy; (B) Cy3-fluorescence light microscopy; (C) transfection efficiency.

Cy3 is a red fluorescent molecule with an excitation wavelength of 480 or 550 nm. The red fluorophore could be seen in the cells successfully transfected with siRNA under the fluorescent microscope (Fig. 1A and B). The transfection efficiency was measured by FCM. Fig. 1C showed that the transfection efficiency was 96.47%. We found that the most optimal transfection concentration of siRNA was 50 nM and the most optimal transfection dosage of Lipo was 5 μl/2 ml after screening the different concentrations of siRNA and Lipo.

### Screening for the most optimal sequence of COX-2 siRNAs silencing COX-2 gene expression in Capan-2 cells

**Figure 2:**
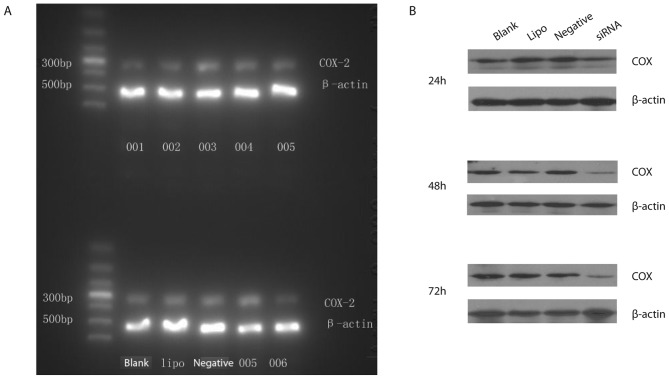
The effect of six different siRNA sequences on silencing the expression of COX-2 mRNA and COX-2 protein in Capan-2 cells. (A) Effect of siRNA on the expression of COX-2 mRNA; (B) effect of siRNA on the expression of COX-2 protein.

Six sequences of COX-2 siRNAs were used to determine the silencing efficiency of COX-2 siRNAs on COX-2 mRNA expression in Capan-2 cells. COX-2 siRNA001, COX-2 siRNA002 and COX-2 siRNA006 were found to be the most optimal sequences. However, COX-2 siRNA003, COX-2 siRNA004 and COX-2 siRNA005 were the invalid sequences (Fig. 2A). COX-2 siRNA006 was found to have the most powerful silencing effect on COX-2 gene. The silencing efficiency of COX-2 siRNA006 was significantly higher than the blank control group (P<0.05). The silencing efficiency of COX-2 siRNA006 on COX-2 mRNA was 73% at 24 h after transfection. Moreover, protein expressions of COX-2 were down-regulated by 67 and 61% respectively at 48 and 72 h after COX-2 siRNA006 transfection. However, there was no significant inhibition effect on protein expression of COX-2 at 24 h after COX-2 siRNA006 transfection. There were no significant differences among the Lipo control group, negative control group and blank control group (P>0.05) (Fig. 2B).

### Cell viability of Capan-2 cells treated with COX-2 siRNA006 at different time points

**Figure 3:**
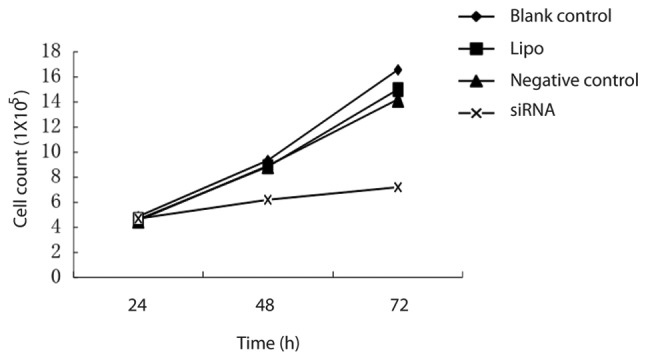
The effect of COX-2 siRNA on the growth curve of Capan-2 cells.

There was no significant difference in cell viability among the different groups at 24 h after transfection (P<0.05). Cell viability of Capan-2 cells in the COX-2 siRNA006 group was significantly decreased at 48 h after transfection. The inhibition rate of cell proliferation was 35.48 and 56.32% respectively at 48 and 72 h after transfection. However, there was no significant difference between the negative group and the Lipo control group (Fig. 3).

### The influence of COX-2 siRNA006 on cell apoptosis of Capan-2 cells

**Figure 4:**
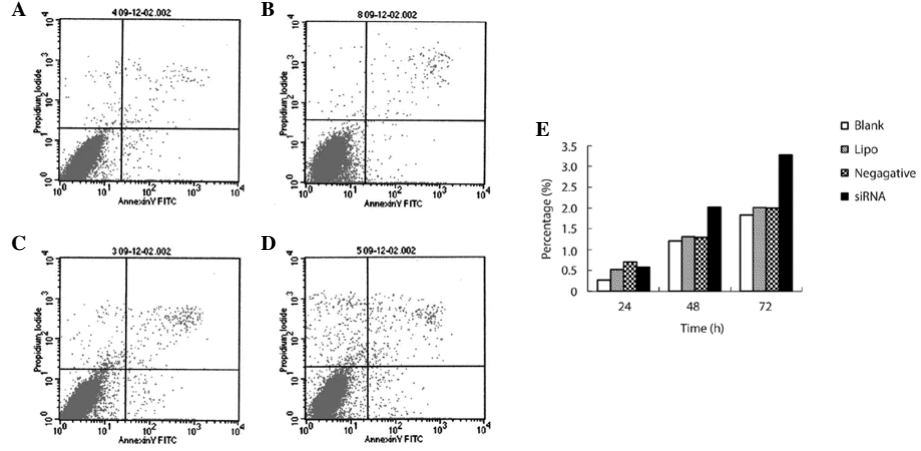
The effect of COX-2 siRNA on apoptosis of Capan-2 cells was tested by FCM. (A) Blank control; (B) Lipo control; (C) negative control; (D) COX-2 siRNA; (E) apoptosis of Capan-2 cells in 24, 48 and 72 h.

The apoptotic cells were increased as the culture time of cells transfected with COX-2 siRNA006 was increased (P<0.05). This was more obvious at 48 h after transfection. There was a significant difference between the COX-2 siRNA group and the negative control group (P<0.05). There was a significant difference between the COX-2 siRNA group and the Lipo control group (P<0.05). However, there were no significant difference among negative control group, the Lipo control group and the blank control group (Table I and Fig. 4).

### The influence of COX-2 siRNA006 on the cell cycle of Capan-2 cells

**Figure 5:**
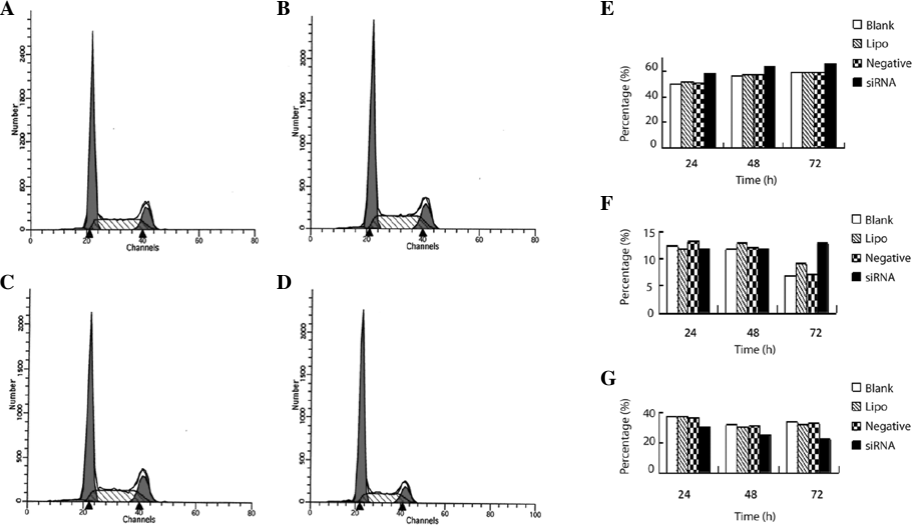
The cell cycle of Capan-2 cells was tested by FCM. (A) Negative control; (B) blank control; (C) Lipo control; (D) COX-2 siRNA; (E) G0-G1 phase; (F) G2-M phase; (G) S phase.

The cells in G0/G1 phase were significantly increased as the culture time of cells transfected with COX-2 siRNA006 increased (P<0.05). However, the cells in the S phase were significantly decreased (P<0.05) (Table II and Fig. 5).

### The influence of COX-2 siRNA on subcutaneous tumorigenicity of Capan-2 cells in nude mice

**Figure 6:**
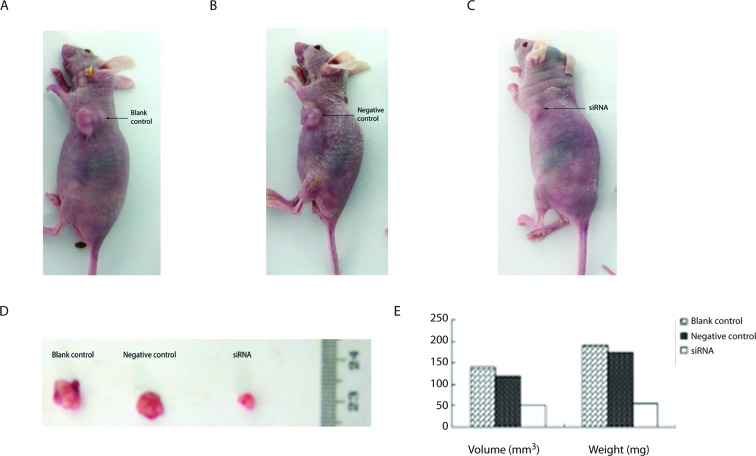
The effect of COX-2-siRNA on the tumor growth of subcutaneous implantation in nude mice. (A) COX-2-siRNA; (B) negative control; (C) blank control; (D) size of the tumor in groups; (E) effect of COX-2 siRNA on the volume and weight of the tumor.

Subcutaneous xenografts could be seen in the control group and the negative control group about 1 week after inoculation. However, those could not be seen in COX-2 siRNA group although subcutaneous xenografts could be seen in COX-2 siRNA group about 2 weeks after inoculation. The xenografts grew significantly more slowly in the COX-2 siRNA group than those in the blank control group and the negative control group (Fig. 6A-C). The size of the xenografts in the COX-2 siRNA group were significantly smaller than those in the blank control group and the negative control group (P<0.05). However, there was no significant difference between the blank control group and the negative control group (P>0.05). Nude mice were sacrificed by cervical dislocation. The xenografts were peeled off and weighed. The data show that the mean weight of xenografts in the COX-2 siRNA group were significantly lower than those in the blank control group and negative control group (P<0.05) (Table III and Fig. 6D-E).

## Discussion

COX-2 could play a role in tumorigenesis via various pathways. First, cyclooxygenase is an enzyme with a dual function of both a cyclooxydase and a peroxidase. The latter could directly activate an oncogene or inhibit mutation of tumor suppressor gene. Second, as COX-derived metabolites, prostaglandins (PGs), especially PGE2 could promote proliferation of normal cells and cancer cells (5–7). Third, overexpression of COX-2 could prolong the lifetime of cancer cells and inhibit cell apoptosis. Fourth, the expression of COX-2 lead to the production of PGE2. The activation of the cell membrane receptor of PGE2 could increase intracellular cAMP, which could induce the synthesis of vascular endothelial growth factor (VEGF). Thus the increased VEGF could promote neovascularization (8). Fifth, the increased PGE2 could inhibit the activation of natural killer cells and cytotoxic T cells, the production of TNF and IL-15, hyperplasia of T cells and B cells, and induce the production of IL-10 which is an immunosuppressive cytokine. This may lead to down-regulated surveillant immunity and cytolytic activity. Therefore, cancer cells could escape immunologic surveillance; Sixth, expression of COX-2 could enhance the migration and invasion of cancer cells, which is related to the direct up-regulation of the expression of matrix metalloproteinase urokinase-type plasminogen activator by COX-2 (9).

COX-2 is not expressed in the exocrine tissue of the normal pancreas, but is slightly expressed in islet cells of pancreas. Many previous studies showed that the expression of COX-2 in pancreatic cancer tissue was markedly increased (2,10,11). Tucker *et al* reported that the expression of COX-2 mRNA in pancreatic cancer tissue was higher by 60 times than that in adjacent non-tumor pancreatic tissue (12). The expression of COX-2 was notably increased in some human pancreatic cancer cell lines (13). However, the mechanism is still unclear. Our previous studies found that the high expression of COX-2 was related to the high expression of catalyst component of telomerase, hTERT (3,4). That indicated that COX-2 played a crucial role in the tumorigenesis, development and metastasis of pancreatic cancer, which suggests that COX-2 was one of the important targets of gene therapy in pancreatic cancer.

The COX-2 inhibitor could inhibit proliferation of pancreatic cancer cells via down-regulation of the expression of COX-2. However, why do we apply RNAi targeting COX-2 gene to inhibit proliferation of pancreatic cancer cells? A great deal of epidemiological data showed that although long-term use of NSAID may decrease the risk of cancer, it may lead to complications of the gastrointestinal tract, renal, cardiovascular and cerebrovascular system. In addition, the inhibition rate of cell proliferation on pancreatic cancer by the COX-2 inhibitor was limited with a range of 40–62% depending on the dosage of COX-2 inhibitor (14,15). However, the inhibition rate of cell proliferation was increased only by about 10% when the dosage of COX-2 inhibitor was doubled (16). Moreover, the risk of cardiovascular events may be increased when the dosage of COX-2 inhibitor was increased. COX-2 selective inhibitors or COX-2 non-selective inhibitors applied in the previous studies all post-translationally regulated COX-2 expression with a dose-dependent COX-2 inhibition effect and limited inhibition efficiency. As the COX-2 inhibitor does not result in specific inhibition, it more or less inhibited COX-1 which maintained the normal physiological function in body. Therefore, it may increase the risk of hypertension and cardiovascular diseases (17). The safety of COX-2 inhibitor in anticancer research needs further clinical investigation.

RNAi could be used to promote cell apoptosis, inhibit proliferation of cancer cells, invasion and metastasis of cancer and drug resistance by suppressing the expression of oncogene and genes that are related to carcinogenesis and development (3,4). RNAi has become a powerful tool in cancer research as it has the advantage of good reliability, high specificity, low cytotoxicity and long and strong effect so on. However, the key point of its successful application is that the high efficiency silencing sequence (silencing rate >70%) and high-throughput vector could be screened in advance.

Our data showed that liposome Lipofactamine 2000 had as high as 96.47% of a transfection rate in Capan-2 cancer cells. In the meanwhile, COX-2 siRNA006, the most efficient silencing sequence was screened from six COX-2 siRNAs sequences. COX-2 siRNA006 targeting COX-2 gene was used to investigate its effect on cell proliferation, cell cycle and cell apoptosis in Capan-2 pancreatic cancer cells. We found that there was no significant difference in cell viability among different groups at 24 h after transfection. Cell viability of Capan-2 cells in the COX-2 siRNA006 group was significantly decreased at 48 h after transfection. The inhibition rate of cell proliferation was 35.48 and 56.32% respectively at 48 and 72 h after transfection. The cells in G0/G1 phase were significantly increased as the culture time of cells transfected with COX-2 siRNA006 was increased. However, the cells in the S phase were significantly decreased. The apoptotic cells were increased as the culture time of cells transfected with COX-2 siRNA006 was increased.

To further verify the effect of COX-2 siRNA silencing COX-2 gene on cell growth of Capan-2 human pancreatic cancer cells, Capan-2 cells transfected with COX-2 siRNA were subcutaneously inoculated into BALB/c-nu/nu nude mice to investigate the effect of COX-2 siRNA on tumorigenicity of Capan-2 cells. Our data showed that COX-2 siRNA could significantly inhibit the tumorigenicity of Capan-2 human pancreatic cancer cells in nude mice.

The above mentioned data *in vitro* and *in vivo* all indicated that COX-2 siRNA could silence expression of the COX-2 gene in Capan-2 cells and influence cell proliferation, cell cycle, cell apoptosis and tumorigenicity of Capan-2 cells, which may supply gene therapy of pancreatic cancer in clinical practice with therapeutic target and theoretical evidence.

## Figures and Tables

**Table I:** The effect of COX-2-siRNA on apoptosis of Capan-2 cells (n=3).

Group	Time (h)	Apoptosis (%)
COX-2 siRNA	24	0.58±0.32
	48	2.03±0.07^a^
	72	3.27±0.29^a^
Negative control	24	0.70±0.16
	48	1.29±0.23
	72	1.99±0.23
Lipo control	24	0.52±0.41
	48	1.31±0.15
	72	2.01±0.21
Blank control	24	0.27±0.28
	48	1.20±0.16
	72	1.83±0.28

a P<0.01 vs. blank control.

**Table II:** The influence of COX-2 siRNA006 on the cell cycle of Capan-2 cells (%) (n=3).

Group	Culture time (h)	G0/G1	G2/M	S
COX-2 siRNA	24	58.03±1.72^a^	11.70±1.19	30.27±0.53^b^
	48	63.31±1.92^a^	11.81±1.72	24.87±1.03^a^
	72	65.66±0.56^a^	12.81±3.22^a^	22.2±2.24^b^
Negative control	24	50.63±0.68	13.32±0.64	36.04±1.09
	48	57.08±0.47	12.09±1.58	30.83±1.53
	72	59.53±3.19	7.08±1.88	33.06±2.11
Lipo control	24	51.38±3.62	11.66±2.61	36.95±1.35
	48	57.09±3.19	12.88±0.49	30.08±2.68
	72	58.97±3.15	9.06±0.91	31.97±2.41
Blank control	24	49.92±3.54	12.46±1.35	37.61±2.53
	48	56.75±3.66	11.70±3.30	31.56±2.97
	72	59.21±2.82	6.76±2.21	34.03±4.50

a P<0.05 vs. blank control;

b P<0.01 vs. blank control.

**Table III:** The effect of COX-2 siRNA on the volume and weight of subcutaneous xenografts in nude mice.

Group	Volume (mm^3^)	Weight (mg)
COX-2 siRNA	50.00±0.01^a^	54.70±5.35^a^
Negative control	119.30±0.02	175.53±14.36
Blank control	141.00±0.05	191.76±19.25

a P<0.05 vs. blank control.
